# Prevention of Caries and Dental Erosion by Fluorides—A Critical Discussion Based on Physico-Chemical Data and Principles

**DOI:** 10.3390/dj10010006

**Published:** 2022-01-05

**Authors:** Matthias Epple, Joachim Enax, Frederic Meyer

**Affiliations:** 1 Inorganic Chemistry and Center for Nanointegration Duisburg-Essen (CeNIDE), University of Duisburg-Essen, Universitaetsstr. 5-7, 45117 Essen, Germany; 2 Dr. Kurt Wolff GmbH & Co. KG, Research Department, Johanneswerkstr. 34-36, 33611 Bielefeld, Germany; joachim.enax@drwolffgroup.com (J.E.); frederic.meyer@drwolffgroup.com (F.M.)

**Keywords:** caries, enamel, dental erosion, fluoride, surface characterization, teeth

## Abstract

Dental erosion is a common problem in dentistry. It is defined as the loss of tooth mineral by the attack of acids that do not result from caries. From a physico-chemical point of view, the nature of the corroding acids only plays a minor role. A protective effect of fluorides, to prevent caries and dental erosion, is frequently claimed in the literature. The proposed modes of action of fluorides include, for example, the formation of an acid-resistant fluoride-rich surface layer and a fluoride-induced surface hardening of the tooth surface. We performed a comprehensive literature study on the available data on the interaction between fluoride and tooth surfaces (e.g., by toothpastes or mouthwashes). These data are discussed in the light of general chemical considerations on fluoride incorporation and the acid solubility of teeth. The analytical techniques available to address this question are presented and discussed with respect to their capabilities. In summary, the amount of fluoride that is incorporated into teeth is very low (a few µg mm^−2^), and is unlikely to protect a tooth against an attack by acids, be it from acidic agents (erosion) or from acid-producing cariogenic bacteria.

## 1. Introduction

In this review, we discuss the physico-chemical aspects of acidic attack on teeth, both from acidic foods and beverages (dental erosion), and from acid-producing caries bacteria. For this, the available literature was mainly analyzed for the analytical aspects of these effects, i.e., studies in which teeth were subjected to an acidic attack and then further analyzed. We have focused on the action of fluoride, which has a well-established ability to prevent caries. A number of claims that have been made in the literature are discussed, i.e., the incorporation of fluoride into the outer enamel as an acid-protective layer, the hardening of the tooth surface by the incorporation of fluoride, and a bactericidal effect of fluoride.

Teeth consist of calcium phosphate in the form of hydroxyapatite, Ca_5_(PO_4_)_3_(OH) [1,2]. The outer part of teeth, the enamel, contains needle-like, micrometer-sized apatite crystals, whereas the interior, the dentin, contains nanoscopic apatite crystals, similar to those in bone [3]. The biological function of enamel is to cut and masticate, promoted by its high hardness. Consequently, the mineral content in enamel is very high (about 97 wt.%) to ensure its hardness. This hardness is not only due to the mineral content, but also to the complex hierarchical arrangement of the hydroxyapatite needles in enamel [1,2,4]. The density of enamel is 2.6 to 2.8 g cm^−3^ [5], i.e., about 80–85% of crystalline hydroxyapatite (3.18 g cm^−3^), underscoring its high mineral content, but also some degree of porosity.

For the following considerations, it is important to note that the biological apatite in teeth is not a pure (i.e., stoichiometric) hydroxyapatite, but is a so-called biological apatite or bioapatite. This bioapatite contains ionic substitutions for calcium, phosphate, and hydroxide, mainly carbonate CO_3_^2−^ and hydrogen phosphate HPO_4_^2−^ in the phosphate positions, constituting a few weight percent [6,7,8,9,10]. This influences the overall stoichiometry and the acid solubility of the mineral. In general, bioapatite has a higher solubility than stoichiometric hydroxyapatite, due to the presence of carbonate and hydrogen phosphate [11]. The hydroxide group can be substituted by other anions, e.g., fluoride, chloride, or carbonate [1,12]. In the case of fluoride, a full substitution of hydroxide by fluoride would lead to the formation of stoichiometric fluoroapatite, Ca_5_(PO_4_)_3_F, but partial substitutions are also possible. The simultaneous substitution of hydroxide by fluoride and of phosphate by carbonate leads to the formation of mineral francolite, Ca_5−x_(PO_4_, CO_3_)_3_F [13,14]. It is also important to stress that the term “calcium phosphate” is not identical with “hydroxyapatite”, but comprises a whole family of chemical compounds with different formula and chemical compositions [15]. These are often characterized by their molar calcium-to-phosphate ratio. Note that the molar calcium-to-phosphate ratio, which is 5:3 = 1.67 in stoichiometric hydroxyapatite, is usually changed by the aforementioned ionic substitutions [7,16,17,18,19].

## 2. Protection of Teeth against Caries and Erosion by Fluoride-Containing Agents

Enamel is the only part of a healthy tooth that is in contact with the outside. Dentin is protected by either the gingiva or the enamel. If the enamel is eroded by acids (e.g., from food or drinks; denoted as erosive tooth wear [20,21]) or bacterial attack (caries), tooth ache and the progression of tooth damage will proceed into the dentin, leading to heavy pain caused by inflammation of the dental pulp and, finally, tooth loss. It is also important to consider the erosive action of the strongly acidic gastric juice, e.g., during reflux or vomiting [20]. In general, it is, therefore, important to protect enamel by regular care and cleaning.

Teeth are regularly attacked by acidic drinks and food, where the acid-soluble hydroxyapatite is dissolved. Subsequently, remineralization from saliva occurs, which, ideally, restores the lost hydroxyapatite. This acidic attack is usually denoted as dental erosion to distinguish it from mechanical abrasion [22,23]. By definition, dental caries and erosion are different processes, as dental caries is a disease caused by acids produced by bacteria and dental erosion is a process caused by acids not from bacteria (e.g., from food). Schlueter et al. recently defined the terms related to erosive tooth wear [21]. From a chemical point of view, there is no principal difference between an attack by acids from food (erosion) and by acids produced by bacteria (caries). However, the duration of these demineralization processes is usually different (erosion: short time scale, due to the presence of acidic beverages, food, or gastric juice [20]; caries: long time scale [24,25], e.g., in biofilms [26,27]).

Toothpaste mainly has the function of mechanically removing dental biofilms and debris (plaque) and promoting tooth remineralization [28,29,30]. Fluoride has already gained prominence for tooth protection at the beginning of the 20th century [31]. It has evolved into a common constituent of toothpaste, mouthwashes, and dental varnishes. Despite a considerable reduction in the last decades, dental caries is still one of the major diseases in humans [32]. An extensive recent review by Walsh et al., entitled “Fluoride toothpastes of different concentration for preventing dental caries”, discusses the known literature at an evidence-based level. In general, fluoride toothpaste prevents caries in children, adolescents, and adults, compared with fluoride-free toothpastes (fluoride concentrations of 1000 to 1250 ppm). The protection increases with increasing fluoride concentration. However, levels of fluoride that are too high may induce fluorosis in young children [24]. The efficiency of fluoride is proven beyond reasonable doubt, but the underlying chemical reactions are still not clear. Regarding clinical studies on fluorides and caries, it is notable that most of the studies (approximately 80%) described by Walsh et al. were published more than 30 years ago. Just 11 clinical studies were published in the past 20 years [24].

Different modes of action of fluoride in the oral cavity have been proposed [33,34]. However, from chemical and analytical viewpoints, the exact in vivo mechanism of fluoride action is not fully understood. This review focuses on the possible interaction of fluo-ride with enamel. Dentin is not considered, and no agents other than fluoride are included in the discussion.

### 2.1. Analytical Approaches to Investigate the Nature of the Outer Tooth Surface

A tooth is a biological object with many variations depending on its origin, i.e., it varies with the individual person, its individual tooth care history, and, of course, the biological species. Bovine and human teeth are usually studied. It must be noted that although bovine tooth enamel is not identical to human tooth enamel in terms of its density and structure [35], it represents a suitable model system for both in vitro and in situ studies [36]. Fully inorganic samples, consisting of sintered and dense hydroxyapatite, have been suggested to study the relevant processes with less variation than biological samples [37]. This is a valuable approach to standardize the effect of tooth treatments, and to avoid the variability in human teeth and the inherent differences between human teeth and bovine teeth. However, it is unclear whether a sintered solid block of hydroxyapatite has the same properties as a less dense and partially porous sample of tooth enamel, also with respect to the lower acid solubility of sintered hydroxyapatite in comparison to biological apatite that contains carbonate [11]. It is likely that the acid resistance of a sintered hydroxyapatite block is higher than that of biological apatite [11,38,39,40].

A tooth in the mouth is always in a dynamic environment. Saliva, bacteria and other microorganisms, proteins (pellicle), biofilms, etc., all exert their diverse actions. It is difficult to model this environment in an experimental setup, due to its high complexity. However, a reduction in complexity is necessary to identify the causes of any observed effects.

Concerning the tooth surface, it has been shown that the major processes occur at the micro- and nanometer scales. Thus, sensitive methods are necessary to analyze the tooth surface, possible changes upon fluoride exposure, and its erosion after acidic attack. Different analytical methods can be used to analyze tooth surfaces (Table 1).

### 2.2. Formation of a Protecting Fluoride-Rich Layer on the Surface of Teeth

In principle, fluoride ions can be incorporated into the hydroxyapatite structure to form a partially substituted or stoichiometric fluoroapatite, Ca_5_(PO_4_)_3_F. Another fluoride-rich phase is mineral fluorite (not to be confused with fluoride), i.e., CaF_2_, which can precipitate from aqueous solutions containing calcium and fluoride ions. At neutral pH, CaF_2_ is more soluble than fluoroapatite, but this can change at lower pH [28]. Stoichiometric hydroxyapatite is slightly more soluble than stoichiometric fluoroapatite, due to the exchange of hydroxide by fluoride, with the critical pH values reported as 5.5 for hydroxyapatite and 4.5 for fluoroapatite [41]. Note that the critical pH for tooth erosion is not a fixed number, as it depends on the chemical nature of the attacking acidic solution. In general, the presence of phosphate and calcium in the solution lowers the critical pH (i.e. prevents dissolution) because the solution equilibrium of hydroxyapatite is pushed towards the solid phase [20].

The common fluoride sources in dental care are sodium fluoride (NaF), sodium monofluorophosphate (SMFP), amine fluorides (AmF), stannous (II) fluoride (SnF_2_), and titanium fluoride (TiF_4_) [28,29,42,43,44]. They can be applied, for example, as toothpastes, mouthwashes, gels, or varnishes. Note that fluoride ions can be inactivated by calcium-containing ingredients in the toothpaste tube, or even the oral cavity, by forming sparingly soluble CaF_2_ [30,45].

Usually, the protective action of fluoride is investigated by subsequent remineralization and erosion experiments (pH cycling), followed by the analysis of the nature and composition of the tooth surface. In vitro studies with extracted bovine or human tooth, and in situ studies have been reported. In order to be efficient, the fluoride-induced effect has to occur relatively fast, within only 2–3 min, under physiological conditions in the oral cavity, i.e., during tooth brushing. The amount of fluoride toothpaste applied is also important. Ten Cate has argued that the prevention of caries by fluoride toothpastes might be negatively influenced by the small brushing heads of electric toothbrushes, i.e., the application of a relatively small amount of toothpaste [46].

De Leeuw has studied the incorporation of fluoride into a hydroxyapatite crystal by molecular modelling (MD) simulations. A crystal of about 2 nm in size was simulated. She found that fluoride was readily incorporated by the exchange of hydroxide by fluoride (the formation of fluoroapatite) in a strongly exothermic reaction. It was also claimed that fluoride prevents the dissolution of tooth mineral to some extent. However, it is questionable if these conclusions can be easily transposed to the in vivo situation, due to the small crystal size in the model and the impossibility to include the pH in the calculations. Another conclusion was the small penetration depth of fluoride into the crystal, i.e., one nanometer or less [47]. Despite all its limitations, to our knowledge, this is the only modelling study of tooth erosion so far.

The effect of fluoride-containing toothpastes on human teeth was studied by Arnold et al. EDX did not show any effect of fluoride treatment on the elemental composition of the teeth, probably due to the low sensitivity of EDX. By chemical analysis, following surface etching, the authors showed that the fluoride content of the tooth surface had increased by brushing, from about 3.5 to 5.15 µg mm^−2^, i.e., by a factor of two to three. However, this is still a very small fluoride concentration, which indicates very limited incorporation of fluoride into the tooth surface. Nevertheless, the teeth were protected, to some extent, from erosion by fluoridation, but the effect was about 25% at most [48]. Hannig et al. also did not detect fluoride in bacterial biofilms after fluoridation, followed by an in situ study [36]. Lelli et al. did not detect fluoride by EDX after the treatment of extracted human teeth with fluoride toothpaste [23]. These reports underscore the limited sensitivity of EDX and the generally low fluoride concentration on the tooth surface. However, Scholz et al. were able to detect fluoride in teeth by EDX, possibly due to the higher concentration that was applied with fluoride gels, rather than with regular fluoride toothpastes [49].

Scholz et al. have studied fluoride precipitation on human enamel by the immersion of human teeth into different solutions after immersion in human saliva for 120 min for pellicle formation. This is an important point, as freshly prepared, “clean” teeth may react differently with fluoride-containing agents because their surface is not coated by proteins and bacteria. Fluoride-containing gels (NaF, amine fluoride) were applied for 60 s to teeth with and without pellicle (fluoride concentration: 12,500 ppm; pH: 4.75 or 7.0). The fluoride concentration was measured by EDX. Surprisingly, the influence of the pellicle was not significant. The most important parameter was the pH value; acidic fluoride-containing gels led to much higher fluoride incorporation (10–15 at.% F) than neutral fluoride-containing gels (0.1 at.% F). The latter resulted in a fluoride uptake of the order of fluoride-free gels (0 to 0.1 at.% F), i.e., close to zero. A layer of CaF_2_ (also in globules), with a thickness of 400 to 610 nm, was found by SEM/EDX for acidic gels; however, the chemical nature of the fluoride-containing phase (CaF_2_) was not shown by crystallographic methods. Nevertheless, the high fluoride level (up to 15 at.%) cannot be explained by fluoroapatite only [49].

Gerth et al. studied the chemical composition of synthetic hydroxyapatite powder and ground enamel after fluoride treatment by XPS, MAS-NMR, and Raman spectroscopy. They postulated a three-layer structure of CaF_2_, Ca(OH)_2_, and fluoroapatite [50]. However, it is chemically difficult to perceive that a highly basic and reasonably well-soluble layer of Ca(OH)_2_ could exist on the surface of teeth, given the frequent exposition to acidic fluids. Without crystallographic evidence, these claims must be viewed very critically.

Similar experiments were conducted by Sternitzke et al. Dispersions of hydroxyapatite were subjected to fluoride ion treatment at different pH. They found a considerable uptake of fluoride, higher at low pH (6.5) than at high pH (9.5), at a depth of up to 20 nm. The chemical nature of the fluoride phase was only derived from stoichiometric considerations (no crystallographic analyses). It was postulated that CaF_2_ might have precipitated first, but that it had converted to fluoroapatite during the 28-day incubation [51].

Müller et al. studied the chemical composition of hydroxyapatite model surfaces (sintered, crystalline hydroxyapatite [37]) by XPS in combination with ion ablation [41]. They treated the hydroxyapatite objects with NaF or AmF for 5 min. The fact that the objects were not rinsed before subsequent analysis in the dried state is noteworthy, as it leaves undissolved fluoride salts on the object’s surface. They found a penetration of fluoride into the sample of the order of 5–20 nm (pH 6.2, NaF) or 50 nm (pH 4.2, AmF). The surface nature was described as a combination of CaF_2_ (outer surface), Ca(OH)_2_ (below), and fluoroapatite (further below), following the model of Gerth et al. [50]. However, this model was only based on the elemental distribution and not on crystallographic identifications. The article concludes with the following remarkable statement: “It has to be asked whether such narrow Ca(OH)_2_ and FAp layers really can act as protective layers for the enamel” [41].

Loskill et al. treated sintered hydroxyapatite objects with fluoride (NaF, 1000 ppm, pH 9) and found a concentration of fluoride of about 1.5 at.% at a depth of 10–30 nm (by XPS and ion ablation) [52].

Ganss et al. studied the effects of NaF, SnF_2_, and amine fluoride on tooth remineralization in an in situ study on human teeth. NaF reduced the erosion by 19% compared with a fluoride-free control, whereas AmF/NaF/SnF_2_ reduced it by 67% [53]. The protective effect of fluoride was confirmed, but the presence of SnF_2_ may have led to the formation of a protective layer of acid-resistant tin oxide/hydroxide.

Lee at al. treated human teeth with fluoride strips (a gel with 1450 ppm fluoride) or fluoride toothpaste (1450 ppm) and subjected them to pH cycling. The tooth surface was then investigated by XPS depth profiling (with argon ion etching). They did not find fluoride in the untreated control samples (before de-/remineralization and fluoride treatment), indicating that fluoride incorporation into the teeth after regular toothpaste treatment is low. The fluoride treatment protected the teeth from demineralization. The authors did not convert the depth profiling into a nanometer scale, but they postulated an outer layer of CaF_2_, followed by fluoroapatite for the fluoride strips, which was in contrast to toothpaste-treated teeth, where they found a much thinner surface layer of fluoroapatite. However, it is questionable whether the treatment of teeth for 1–24 h with a fluoride-containing gel (“fluoride strip”) is relevant for any practical situation [54].

Lelli et al. studied the tooth surface after fluoride treatment (using extracted human teeth and fluoride toothpaste). Notably, they did not find any other phase than apatite (note that hydroxyapatite and fluoroapatite are practically indistinguishable by XRD). In particular, they did not find evidence for the presence of CaF_2_. Interestingly, the crystallinity of the tooth surface after fluoride treatment was higher than before, indicating better remineralization of the tooth surface to a fluoride-containing apatite [23].

Hjortsjö et al. incubated human teeth in solutions of HF, SnF_2_, TiF_4_, and NaF, under strongly acidic conditions (pH = 1.6 to 3.1). By SEM and EDX, they found fluoride-rich films with a thickness of 200 to 800 nm, depending on the fluoride-containing agent (incubation time: 10 min). The fluoride content was the highest for HF and acidified NaF (which is essentially the same as HF), i.e., between 7.3 and 14 wt.%. The application of NaF (not acidified), SnF_2_, and TiF_4_ led to much lower fluoride contents, between 0.4 and 0.7 wt.%. For SnF_2_ and TiF_2_, considerable concentrations of these metals were found, but with a low fluoride content. This points to precipitated tin and titanium oxides/hydroxides that act as an acid-protective layer. There were also strong indications for the formation of globular CaF_2_ particles on the tooth surface for HF and acidified NaF, based on the EDX spectra (molar ratio of Ca:F of about 1:2). Thus, at low pH, the tooth surface dissolves and releases calcium ions that reprecipitate as CaF_2_ [55]. It is highly questionable whether these results can be transposed to any real situation because the applied pH values were very low. The inherent toxicity of HF that is increasingly formed below a pH of 3.5 would prohibit any such tooth treatment.

Faidt et al. studied the kinetics of fluoride uptake into sintered hydroxyapatite blocks. The penetration of fluoride was studied by XPS in combination with laser ablation. The objects were subjected to 500 ppm fluoride at pH 5.5 for time periods between 15 s and 5 min. Fluoride was detected at a depth of 40 nm after 5 min. However, the changes after 3 min were only minor, i.e., the equilibrium had mostly been reached at this time. At the surface (first few nm), the fluoride content was equivalent to a fluoride substitution of about 62% in hydroxyapatite. The authors postulated a different rate of fluoride uptake as a function of the crystallographic face of a given hydroxyapatite crystal (but without providing experimental evidence for this assumption) [56].

Tasios et al. published a meta-review on orthodontic enamel demineralization. Fluoride varnish may prevent so-called white spot lesions (WSL), i.e., demineralized spots on a tooth, but the quality of evidence was assessed as low due to the poor quality of the studies [57]. Sardana et al. came to the same conclusion in a meta-review on the same topic. The protecting effect of fluoride may be there, but the statistical evidence is low due to insufficient study quality [58].

Zanatta et al. published a meta-review on the protective effect of fluoride compounds on erosive tooth wear. In general, the quality of the evidence was low to moderate, but the protective effect of fluoride can be safely acknowledged. The published studies were very heterogeneous, and more research was deemed necessary [59].

Amaechi et al. studied the effect of fluoride-containing oral care gels on the remineralization of initial caries in a pH cycling study. Bovine teeth were first demineralized to produce artificial caries lesions. They were then remineralized with a fluoride-based gel (12,500 ppm fluoride), and with artificial saliva (3 min). The mineral loss/gain was assessed by radiography (X-ray density). The remineralization in the presence of the fluoride gel was much stronger than that in the presence of fluoride-free artificial saliva [60]. Amaechi et al. also exposed human teeth to fluoride toothpaste (500 ppm amine fluoride) in an in situ study. The fluoride treatment led to significant (but not homogenous) remineralization of the initially acid-induced caries lesions (optical image analysis) after 14 days [61].

Thus, the chemical nature of the outer layer of teeth is still a subject of discussion. It is clear from the published studies discussed above that the tooth surface does not consist of one chemical compound only, but of a number of layers with a thickness of a few dozen nanometers each. This makes the results of all the methods with limited depth resolution difficult to interpret, as they provide only integral data. This applies, e.g., to EDX, XRD, and all elemental analysis methods that are based on chemical dissolution of the outermost tooth layer. On the other hand, purely surface-sensitive methods, such as SEM and AFM, are not sensitive for “buried” compounds below the surface.

The actual content of fluoride in a tooth after fluoride treatment is difficult to assess because there is clearly a gradient from the outside (high fluoride content) to the interior (where the fluoride content is almost zero). Thus, it depends on the thickness of the surface layer that is analyzed, as this forms the volume that is used to compute the concentration. From the above data, it follows that the total amount of fluoride that is incorporated into the tooth surface is of the order of a few µg mm^−2^.

It must be emphasized that without a proper crystallographic investigation by X-ray diffraction (XRD) or electron diffraction (ED), most conclusions on the surface chemistry (and crystallography) of teeth remain highly speculative. For the microscopic analysis of the nature of a tooth surface, SEM, TEM, XPS, and AFM have been applied (see Table 1). When it comes to the chemical nature of a tooth surface at the nanometer scale, XPS coupled with ion ablation is the method of choice because EDX lacks the necessary resolution and sensitivity. However, XPS only provides indirect information on the chemical compounds in and on the tooth surface. The stoichiometry is derived from the ratio of elements, which is an ambiguous approach. This cannot replace crystallographic analysis, which is, however, difficult due to the required nanometer resolution.

### 2.3. Protective Action of Fluoride against Acidic Attack: Dissolution Studies

The protective action of fluoride could be due to chemical and biological effects. We will discuss the chemical effects first, i.e., dissolution studies that mimic dental erosion.

Kwon et al. studied the dissolution of hydroxyapatite single crystals by in situ AFM in the presence of fluoride (0.2 to 200 ppm) at pH 6. The surface erosion rate was clearly changed by the presence of fluoride. This may indicate slower dissolution, but it is not clear whether these observations can be transposed to human tooth erosion due to the different chemical composition (sintered hydroxyapatite vs. bioapatite) [62].

Parker et al. performed an in situ AFM study on the dissolution of bovine enamel by acids (pH 3). A previous treatment with fluoride extended only 10 to 20 nm into the enamel surface, so the dissolution was only postponed by a few seconds [63].

Faidt et al. studied the protective effect of fluoride on the acid resistance of hydroxyapatite, using sintered hydroxyapatite blocks as the test specimen. Fluoridation was performed with NaF (500 ppm) at pH 6 for 5 min. The dissolution was studied by in situ AFM at pH 4.5. Remarkably, they found “quickly etching areas” and “slowly etching areas”, attributed to the different crystallographic orientations of the hydroxyapatite grains on the polished sample surface. The fluoride content of the hydroxyapatite surface was decreased after 1770 s, to about 50% of the original value (measured by XPS), indicating dissolution of the fluoride-containing layer and a loss of fluoride. Fluoridation inhibited the surface dissolution for about 330 s, but, after that time, the dissolution was as fast as without fluoridation [64].

### 2.4. Protective Action of Fluoride: Biological Considerations

The possible biological effects of fluoride that have been suggested are the bactericidal action of fluoride and reduced bacterial adhesion on a fluoride-treated tooth surface. However, the antibacterial action of fluoride compounds is mainly determined by the antibacterial counter ion, e.g., amine and stannous ions [65]. Some studies have shown that fluoride itself is antibacterial because it inhibits the bacterial enzyme enolase; enolase is important for glycolysis [66]. Nevertheless, it can be questioned whether fluoride ions applied in low concentrations for a few minutes during tooth brushing are effective and able to penetrate and interact with oral biofilms [27,67]. An innovative idea is the application of laser irradiation to control biofilm growth on the tooth surface [68].

Hannig et al. studied the effect of mouthwashes with and without fluoride on bacterial adhesion in an in situ study with bovine enamel. Bacterial adhesion was reduced by both chlorhexidine and amine fluorides [36]. However, amine fluorides have a distinct antibacterial effect, which is not due to fluoride, but to the organic part (the amine) [65].

Loskill et al. studied the effect of bacterial adhesion on sintered hydroxyapatite objects after fluoride treatment (NaF, 1000 ppm, pH 9). The bacterial adhesion was reduced by a factor of about two. The possible reasons for this were complex, but the adhesion force of bacteria (as measured by AFM) was reduced after surface fluoridation [52].

Kirsch et al. studied the effect of fluoride treatment on the bacterial colonization of bovine teeth. The teeth were incubated with fluoride (500 ppm) from various sources (NaF, sodium monofluorophosphate, amine fluoride, and SnF_2_, as well as SnCl_2_ for comparison) for 1 min. The bacterial colonization with relevant strains (in situ study) was analyzed by optical microscopy. In general, neither NaF nor sodium monofluorophosphate had a significant effect, whereas amine fluoride and SnF_2_/SnCl_2_ significantly reduced the bacterial adhesion. This can be explained by the antibacterial effect of the amine in amine fluoride, and possibly by a protective effect of the formed tin oxides/hydroxides. The fact that there was no difference between SnF_2_ and SnCl_2_ suggests that fluoride was not responsible for the observed reduction in bacterial adhesion. Furthermore, the solutions of NaF and sodium monofluorophosphate were pH neutral, whereas all the other solutions were acidic (3.5 to 4.5). The effect of a low pH and the resulting surface erosion, followed by reprecipitation, was, therefore, not taken into consideration [69].

### 2.5. Protective Action of Fluoride: Mechanical Considerations

A frequent claim in the commercial promotion of fluoride-containing toothpastes and other treatments is the “hardening” of the tooth structure by the presence and incorporation of fluoride.

Delbem et al. studied the mineral content and microhardness of bovine teeth after pH cycling and toothpaste treatment. They varied the fluoride content in the toothpaste and found a clear increase in microhardness with increasing fluoride content, by a factor of four to five. This effect was found in the outer layer of the teeth up to a depth of about 90 µm, i.e., where de- and remineralization occur. Beyond that depth, all teeth had the same hardness. This effect was strongly correlated with the mineral content of this outer tooth layer; a higher mineral content led to higher surface hardness [70].

Moretto et al. studied demineralization and remineralization in bovine enamel by measuring the microhardness, with and without fluoride treatment. They found a clear increase in tooth hardness and fluoride content (by a factor of about two), and also a reduction in erosion by a factor of two to three after fluoride treatment. They postulated a mechanism where less erosion leads to higher hardness of a tooth [22].

Creeth et al. studied the effect of fluoride-containing toothpastes (2 min brushing) on the erosion and remineralization of bovine teeth in an in situ study (up to 8 h after brushing). They measured the microhardness of enamel before and after erosion, and the subsequent remineralization. The acid resistance of the fluoride-treated teeth was higher than that of the untreated teeth. However, the fluoride-treated teeth were not significantly harder than the untreated teeth. The fluoride content of the fluoride-treated teeth was significantly higher, as studied on microdrill enamel biopsies (100 µm depth). The fluoride content was 1 µg F cm^−2^ for the untreated teeth and 1.5–2.7 µg F cm^−2^ for the fluoride-treated teeth. Thus, the fluoride uptake was significant, but amounted to a very low overall concentration. The authors conclude that only a very small fraction of the applied fluoride is taken up by the teeth during brushing [71].

These results are in line with the considerations that tooth enamel is mainly eroded by organic acids from food, beverages, and gastric juice. Organic acids are typically weak Brønsted acids. Therefore, a substantial fraction of them are present in an undissociated (molecular, uncharged) state that can diffuse faster into ionic solids, such as tooth mineral [20]. This leads to a partially demineralized, softened enamel layer of a few micrometers thick [72]. The presence of fluoride may contribute to the hardening of this layer, but, as shown above, the amount of incorporated fluoride is rather low.

Shark tooth enameloid is an interesting model system because it contains very high amounts of fluoride [73,74]. The hardness of shark tooth enameloid (fluoroapatite; approx. 3.1% fluoride [75]) and human tooth enamel (hydroxyapatite; approx. 0.01% fluoride [76]) is comparable [75]. Consequently, even if it was possible to incorporate high amounts of fluoride into human enamel (which is clearly not the case), the degree of “surface hardening” is not expected to be strong. Ogaard et al. compared the demineralization process of shark tooth enameloid and human enamel in a microradiographic study. They found that that even shark tooth enamel with 30,000 ppm fluoride is dissolved due to cariogenic challenges, and offers only limited protection against caries [77].

## 3. Conclusions

The available literature on this topic is vast, but the main results converge to some basic facts. However, there is a lack of evidence-based comparative studies that support the major conclusions. Here, we draw the following conclusions:

- The protective and remineralizing effect of fluoride has been clearly demonstrated. It is important to note that calcium and phosphate ions are necessary for the action of fluoride (usually derived from saliva).
- At neutral pH, fluoride is incorporated into the outer surface of teeth, to a small extent, but only at the length scale of several tens of nanometers (total fluoride concentration in the range of a few µg F mm^−2^). The protective action of fluoride is, therefore, not due to its incorporation into the tooth, but rather to an accelerating effect on the remineralization.
- A treatment with fluoride under acidic conditions leads to partial dissolution (demineralization) of the enamel surface (hydroxyapatite), followed by reprecipitation of fluoride-containing minerals. The thickness of this fluoride-containing layer increases with increasing fluoride concentration, and is favored by a low pH value. It can reach several hundred nanometers.
- The chemical nature of the deposited fluoride-containing phase has not yet been assessed by a proper crystallographic analysis as a function of depth. The formation of CaF_2_ has often been claimed, but has never been proven directly, except for indirect considerations by elemental analysis, XPS, or EDX. Thus, there is an urgent need to identify the deposited mineral phase by crystallographic methods. At very high fluoride concentrations (e.g., fluoride gels; not toothpastes or mouthwashes), the formation of CaF_2_ appears to be likely, but, in most cases, there is (limited) incorporation of fluoride into the enamel hydroxyapatite to likely form fluorohydroxyapatite or francolite. It must be stressed that the chemical nature of bioapatite (carbonate containing and calcium deficient) has not been considered in the reported studies. Most authors assume stoichiometric hydroxyapatite as the tooth mineral phase or simply use the term “apatite”.
- It has been claimed that a surface layer that contains Ca(OH)_2_ is present on teeth. However, it has never been proven, and its presence is very unlikely in the pH cycling environment of a tooth, due to its high acid solubility and comparatively high water solubility (about 1 g L^−1^).
- It is unlikely that the formed fluoride-containing layer can protect the teeth from erosion for more than a few minutes at a pH of 5 or below (as introduced by many beverages). It is simply too thin, too porous, and too easily soluble.
- The use of synthetic hydroxyapatite blocks as model surfaces is important to standardize the experiments but raises questions about the differences between porous tooth enamel consisting of bioapatite and sintered (dense) hydroxyapatite.
- There is no clear evidence for reduced bacterial adsorption on a fluoridated tooth surface. Some effects may be due to the application of amine fluoride, where the amine cation has the main antibacterial effect.
- There are conflicting reports on the increase in tooth hardness after fluoride treatment. In any case, this effect appears to only be present on the outermost enamel surface (several micrometers at most).

## Figures and Tables

**Table 1:** Overview of methods for analyzing tooth surfaces (ordered alphabetically). All methods work with extracted human teeth as well as with bovine teeth; however, they are not applicable directly in the oral cavity.

Analytical Methods	Results
Atomic force microscopy (AFM)	Can probe the surface topography of a tooth with a vertical resolution of 1 nm or better. It can also be applied in situ (i.e., in a time-resolved way) and also on a surface that is immersed in water or another liquid phase.
Electron-backscattered diffraction (EBSD)	Provides information on the crystallographic nature of a surface, e.g., the orientation of individual crystals.
Electron diffraction (ED)	Usually combined with transmission electron microscopy (TEM). It can identify crystallographic phases at the nanometer length scale.
Elemental analysis	Can be performed by, e.g., atomic absorption spectroscopy (AAS), X-ray fluorescence analysis (XRF), inductively coupled plasma mass spectrometry (ICP-MS), or a fluoride-sensitive electrode; all these methods have different accuracy. In the case of teeth, it requires dissolution of the outer tooth layer, e.g., by application of an acid or by mechanical abrasion. It gives an overall value of the surface composition, without information on the chemical constituents, e.g., the solid phases present.
Energy-dispersive X-ray spectroscopy (EDX)	Usually coupled with SEM or TEM. It provides information on the elemental composition of a tooth. Its sensitivity is one percent or less. Fluoride is sometimes difficult to detect, due to the vicinity of its EDX peak to omnipresent oxygen and nitrogen.
Fluorescence microscopy and confocal laser scanning microscopy	Can show the number and vital status (live/dead) of bacteria that are adsorbed on a tooth surface.
Indentation measurements (micro and nano)	Provides information on the hardness of teeth on the micrometer and nanometer length scale.
Microcomputer tomography (µCT)	Provides information on the mineral content and mineral density on the length scale of several micrometers, but not on the crystallographic phase.
Modelling techniques (e.g., molecular dynamics, force field methods)	Difficult to apply to teeth because of the complex nature of teeth and the surrounding liquid (e.g., saliva). This requires the consideration of large systems that are beyond the current computation possibilities.
Scanning electron microscopy (SEM)	Provides the morphology of the tooth surface or of cross-sections at the nanometer scale. Individual crystals can be identified (if present).
Transmission electron microscopy (TEM)	Has a higher resolution than SEM, but requires thin samples, i.e., usually cross-sections of a material with a thickness of about 100 nm.
X-ray photoelectron spectroscopy (XPS)	Highly sensitive method for elemental surface analysis. It also provides information on the oxidation state of an individual element. It can only probe the outer few nanometers of a sample; however, it can be combined with ion ablation techniques (usually by argon ions) to analyze the surface at a depth of several nanometers.
X-ray powder diffraction (XRD)	Crystallographic identification of a solid sample, e.g., the unequivocal identification of calcium phosphate as hydroxyapatite. If applied to a tooth surface, it provides information on a surface layer of several micrometers thick. Thus, it is not suitable to differentiate between different crystal phases (i.e., types of chemical compounds with distinct chemical formula and crystal structure) that are present on the tooth surface in a layered way at the scale of a few micrometers.

## Data Availability

Not applicable.
